# Gender and the length of time since autologous hematopoietic stem cell transplantation—What is their influence on the immune reconstitution in multiple myeloma patients?

**DOI:** 10.1371/journal.pone.0295308

**Published:** 2023-12-07

**Authors:** Natalia Popierz-Rydlewska, Sylwia Merkiel-Pawłowska, Anna Łojko-Dankowska, Mieczysław Komarnicki, Wojciech Chalcarz

**Affiliations:** 1 Team of Food and Nutrition, Department of Dietetics, Faculty of Health Sciences, Poznan University of Physical Education, Poznań, Poland; 2 Department of Haematology and Bone Marrow Transplantation, Poznan University of Medical Sciences, Poznań, Poland; 3 Poznan University of Medical Sciences, Poznań, Poland; 4 Poznan University of Physical Education, Poznań, Poland; University of New Hampshire, UNITED STATES

## Abstract

**Introduction:**

In the literature there is lack of information on the influence of gender and time since autologous hematopoietic stem cell transplantation (HSCT) on the immune reconstitution in multiple myeloma (MM) patients.

**Objective:**

The aim of this study was to assess the diversity of the immune reconstitution according to gender in MM patients after autologous HSCT on the day of the clinic discharge and on the 29th day after discharge, as well as to investigate the changes in the immune system in females and males after staying at home for 28 days.

**Method:**

The studied population comprised 13 females and 13 males after autologous HSCT. On the day of the clinic discharge and on the 29th day after discharge blood samples were taken to analyse 22 immunological parameters. Statistical analysis was performed using STATISTICA 10 StatSoft Poland. For multiple comparisons, the Bonferroni correction was used.

**Results:**

No statistically significant differences were observed in the analysed immunological parameters between the studied females and males with MM on the day of the clinic discharge and on the 29th day after discharge. However, on the 29th day after the clinic discharge compared to the day of the clinic discharge, statistically significant differences were found in 8 immunological parameters among females and 6 immunological parameters among males.

**Conclusion and recommendation:**

Our results indicate that the immune reconstitution is similar but not the same in patients of both genders. Statistically significant differences in the immune response in the studied females and males imply that gender may play a role in the immune reconstitution and that the results obtained in MM patients should be analysed separately in females and males. In order to explain the observed changes in the immune system according to gender, further research should be carried out on a larger population. This would most probably make it possible to find their clinical application.

## Introduction

It is well-known that the post-transplant immune reconstitution is a multistage process, and although granulocytopoiesis reconstitutes within 2–3 weeks after the transplantation, full recovery may last more than ten years [1–5]. Factors which influence the rate of the immune recovery include patient’s overall condition, the degree of the thymus function damage, damages caused by the therapy, type of transplantation (autologous or allogeneic), source of hematopoietic cells (from bone marrow, peripheral blood or cord blood) and hematopoietic cell count in the transplant, especially mature lymphocytes [3, 6]. There is lack of information how gender and time since autologous hematopoietic stem cell transplantation (HSCT) influence the immune system reconstitution in MM patients. In order to fill this gap in the literature, the research was conducted to assess the diversity of the immune response according to gender in MM patients after autologous HSCT on the day of the clinic discharge and on the 29th day after discharge, as well as to investigate the changes in the immune system in females and males after staying at home for 28 days.

## Subjects and methods

### Subjects

The studied population comprised 26 MM patients, 13 females and 13 males, after autologous HSCT. The subjects were the patients of the Haematology and Bone Marrow Transplantation Department of the Clinical Hospital of the Poznan University of Medical Sciences, Poland.

This study was approved by the Bioethics Committee of the Poznan University of Medical Sciences, Poland (347/10, 467/11). The study was not a medical experiment and had no influence on the treatment methods.

Once the granulopoiesis reconstitution was observed, that is from the moment when patients may have contact with persons other than hospital staff, the patients were asked whether they would like to take part in the research voluntarily. Each patient was informed about the aim, scope and methods of the research during an individual meeting and about the possibility to withdraw from the study at any time without any consequences. The patients gave written consent to take part in the study. During the time from the day of the clinic discharge until the 29th day after discharge the main researcher maintained contact with each patient by telephone. The study was completed by all patients with MM who wished to engage in the study.

### Methods

Date of birth, domicile, gender, disease, the type of transplantation and the length of time since the diagnosis to the transplantation, as well as the number of days from the transplantation until the day of the clinic discharge were taken from patient charts. Because two of the studied patients underwent two transplantations, both the time since the diagnosis to the first transplantation as well as the time since the diagnosis to the second transplantation were taken into account. The latter time was called the time since the diagnosis to the recent transplantation. After the recent transplantation all the immunological parameters presented in the current article were assessed. The percentage of patients who lived after the first transplantation more than four years was calculated based on the information from patient charts.

Blood samples were taken from the studied patients twice: on the day of the clinic discharge and during a follow-up visit on the 29th day after discharge. We chose the day of discharge instead of a specific post-infusion day because we wanted all patients to meet certain conditions: before discharge each patient must be in good general condition, without signs of infection and with signs of reconstitution of hematopoiesis. Engraftment after hematopoietic stem cell transplantation is defined as an absolute neutrophil count (ANC) greater than 500 cells per microlitre of blood on the first day of three consecutive days and platelet recovery is defined as a platelet count greater than 20,000 cells per microlitre of blood on the first day of seven consecutive days without transfusion support. Engraftment usually occurs between the 10th and the 14th day after stem cell infusion and depends on many factors. Patients in our analysis were discharged when the ANC count was at least 1000 cells per microlitre and were transfusion independent. This is the first day on which the patient, according to the current knowledge, may leave the hospital ward and may continue the treatment at home. Our patients were discharged between 13 and 30 days after infusion (median 18).

Our choice of the 29th day after discharge was related to the expected condition of the patient, but also to some organisational considerations. Usually, after four weeks, the patients’ general condition, nutritional status and peripheral blood parameters improve and they can continue treatment in outpatient clinic.

Blood samples were taken after an overnight fast from the ulnar vein using the S-Sedivette^®^ venous blood collection system produced by Sarstedt, Germany.

BN II nephelometric analyser made by Siemens, Germany, was used to assess serum immunoglobulin A (IgA), immunoglobulin G (IgG) and immunoglobulin M (IgM) concentration. Dimension EXL 200 analyser made by Siemens, Germany, was used to assess serum C-reactive protein (CRP) concentration. Analyser ADVIA 2120i made by Siemens, Germany, was applied to assess blood leukocyte count, lymphocyte count, neutrophil count, eosinophil count, basophil count, monocyte count, the percentages of basophils, eosinophils, granulocytes, monocytes and neutrophils in total leukocyte count. T and B lymphocytes subpopulations, and natural killer (NK) cell subpopulation were assessed by means of flow cytometry using Becton Dickinson monoclonal antibodies, USA, and Becton Dickinson FACS Calibur flow cytometer, USA.

The analyses were done in the laboratories of the Hematology and Bone Marrow Transplantation Department of the Clinical Hospital of the Poznan University of Medical Sciences, Poland.

### Statistical analysis

Statistical analysis was performed using STATISTICA 10 StatSoft Poland. Means, standard deviations, medians, lower quartiles and upper quartiles were calculated. The Shapiro-Wilk statistic was used to test the normality of quantitative variables. Since most of the variables were skewed, the non-parametric Mann-Whitney *U* test was used to investigate the differences between immunological parameters in females and males, whereas the Wilcoxon’s signed-rank test was used to compare immunological parameters on the day of the clinic discharge and on the 29th day after discharge. The level of significance was set at *P*≤0.05. For multiple comparisons, when comparing two genders in two time points—four comparisons in total–the Bonferroni correction was applied. It is calculated by dividing the significance level (α) for a single test by the number of tests (n), that is 0.05:4 = 0.0125. Therefore, the level of significance for these comparisons was set at *P*≤0.0125.

## Results

### Characteristics of the studied patients

Table 1 presents characteristics of the studied MM patients. No statistically significant differences between females and males were observed.

**Table 1:** Characteristics of the studied MM patients.

Variable		Females	Males	*P*
		(n = 13)	(n = 13)	
Age [years]	$\bar{x}\pm \;\text{SD}$	58.6±7.4	56.2±8.9	0.5446
	Me	61.4	58.2	
	Q_1_-Q_3_	54.8–64.4	46.9–63.0	
The length of time since the diagnosis until the first transplantation [months]	$\bar{x}\pm \;\text{SD}$	19.5±4.8	30.0±17.5	0.1191
	Me	17.0	25.0	
	Q_1_-Q_3_	15.0–25.0	19.0–35.0	
The length of time since the diagnosis until the recent transplantation [months]	$\bar{x}\pm \;\text{SD}$	21.9±7.4	31.5±16.6	0.1056
	Me	21.0	26.0	
	Q_1_-Q_3_	17.0–25.0	20.0–35.0	
The number of days from the transplantation until the day of the clinic discharge	$\bar{x}\pm \;\text{SD}$	18.1±3.07	18.2 ±4.59	0.8425
	Me	18.0	17.0	
	Q_1_-Q_3_	16.0–19.0	16.0–19.0	
Percentage of patients who lived after the first transplantation more than 4 years [%]		53.8	76.9	0.2159
Percentage of patients who lived in a given region of Poland [%]	Wielkopolskie	69.2	69.2	-
	Lubuskie	23.1	23.1	
	Zachodnio-pomorskie	7.7	7.7	

$\bar{x}$- mean, SD -standard deviation; Me—median,Q1—lower quartile; Q3—upper quartile; *P*—significance.

Table 2 shows CD34+ cells dose, the number of days from the transplantation until the day of the clinic discharge and number of neutrophils on the day of discharge in each of the studied MM patients.

**Table 2:** CD34+ cells dose, the number of days from the transplantation until the day of the clinic discharge and number of neutrophils on the day of discharge in each of the studied MM patients.

Gender	CD34+ cells dose [x10^6^/kg]	Day of discharge	Number of neutrophils on the day of discharge [10^9^/l]
Female 1	4.055	13	2.170
Female 2	3.750	15	0.990
Female 3	4.590	15	2.290
Female 4	4.720	16	3.543
Female 5	4.170	17	1.620
Female 6	3.700	18	1.770
Female 7	5.050	18	1.780
Female 8	3.510	19	3.040
Female 9	3.820	19	1.400
Female 10	3.275	19	2.030
Female 11	4.170	20	2.240
Female 12	4.480	21	1.590
Female 13	4.540	25	2.520
Male 1	3.740	13	1.390
Male 2	3.500	14	0.750
Male 3	4.390	15	2.060
Male 4	3.310	16	3.290
Male 5	3.310	16	5.100
Male 6	3.990	16	1.950
Male 7	3.350	17	1.788
Male 8	3.000	19	3.090
Male 9	3.510	19	5.000
Male 10	5.210	19	1.950
Male 11	3.850	21	2.030
Male 12	3.950	24	3.472
Male 13	4.320	30	2.810

### The immune reconstitution

Table 3 shows immunological parameters in the studied MM patients on the day of the clinic discharge and on the 29th day after discharge.

**Table 3:** Immunological parameters in the studied MM patients on the day of the clinic discharge and on the 29th day after discharge. The explanation of the significance levels (P1, P2, P3, P4) was given below the table.

Parameter		On the day of the clinic discharge		On the 29th day after discharge		Significance level			
		Females	Males	Females	Males	*P*1	*P*2	*P*3	*P*4
		(n = 13)	(n = 13)	(n = 13)	(n = 13)				
Basophils [10^9^/l]	$\bar{x}\pm \;\text{SD}$	0.03±0.02	0.06±0.07	0.04±0.03	0.05±0.03	0.3107	0.3358	0.4561	0.9375
	Me	0.02	0.04	0.03	0.03				
	Q_1_–Q_3_	0.01–0.04	0.02–0.04	0.02–0.05	0.03–0.06				
Basophils [%]	$\bar{x}\pm \;\text{SD}$	0.9±0.7	1.7±2.8	0.7±0.4	0.9±0.5	0.5446	0.5114	0.4216	0.3882
	Me	0.7	0.8	0.7	0.8				
	Q_1_–Q_3_	0.5–1.2	0.5–1.5	0.4–0.9	0.5–0.9				
CD4(+) T lymphocytes [%]	$\bar{x}\pm \;\text{SD}$	33.5±22.0	28.2±16.4	10.9±4.4	16.1±9.8	0.8801	0.0879	**0.0080**	0.0208
	Me	29.5	24.7	9.5	14.5				
	Q_1_–Q_3_	15.7–48.7	19.3–31.0	8.9–11.2	9.5–16.8				
CD8(+) T lymphocytes [%]	$\bar{x}\pm \;\text{SD}$	43.4±19.8	32.8±21.4	62.5±18.7	52.9±16.3	0.1896	0.1330	0.0409	0.0208
	Me	46.4	26.1	70.4	51.0				
	Q_1_–Q_3_	25.1–58.7	15.0–47.8	56.3–75.8	39.9–68.2				
CD19(+) B lymphocytes [%]	$\bar{x}\pm \;\text{SD}$	1.2±2.8	0.6±0.8	10.9±13.7	11.1±11.6	0.8328	0.7477	**0.0044**	**0.0058**
	Me	0.5	0.5	3.7	10.0				
	Q_1_–Q_3_	0.0–0.6	0.0–0.9	3.5–14.0	3.5–15.0				
CRP [mg/l]	$\bar{x}\pm \;\text{SD}$	6.73±7.21	17.67±17.80	1.76±0.85	7.08±11.46	0.1774	0.0301	0.0357	0.0367
	Me	4.80	5.60	1.77	2.40				
	Q_1_–Q_3_	1.75–8.41	4.56–26.40	1.20–1.90	2.00–3.20				
Eosinophils [10^9^/l]	$\bar{x}\pm \;\text{SD}$	0.03±0.09	0.01±0.01	0.26±0.24	0.24±0.35	0.3358	0.3897	**0.0088**	**0.0015**
	Me	0.01	0.00	0.25	0.11				
	Q_1_–Q_3_	0.00–0.02	0.00–0.01	0.08–0.33	0.09–0.19				
Eosinophils [%]	$\bar{x}\pm \;\text{SD}$	0.9±2.1	0.1±0.2	5.2±4.4	4.5±6.2	0.2226	0.2428	**0.0107**	**0.0015**
	Me	0.2	0.1	3.3	2.5				
	Q_1_–Q_3_	0.1–0.4	0.039–0.18	2.1–7.5	1.4–4.6				
Granulocytes [%]	$\bar{x}\pm \;\text{SD}$	27.7±4.7	66.2±13.8	42.1±5.0	46.5±23.0	0.1000	0.5714	0.1088	**-** *
	Me	26.0	68.0	41.0	58.0				
	Q_1_–Q_3_	24.0–33.0	51.5–79.0	40.0–46.0	20.0–61.5				
IgA [g/l]	$\bar{x}\pm \;\text{SD}$	1.1±1.7	0.6±0.3	0.9±1.8	0.7±0.6	0.8801	0.3164	0.5754	0.5751
	Me	0.4	0.7	0.3	0.6				
	Q_1_–Q_3_	0.3–1.0	0.3–0.8	0.3–0.5	0.2–0.9				
IgG [g/l]	$\bar{x}\pm \;\text{SD}$	5.7±1.4	6.8±3.6	8.0±4.0	9.8–5.2	0.4865	0.5254	0.0367	**0.0033**
	Me	6.0	6.3	7.2	7.9				
	Q_1_–Q_3_	4.3–6.9	4.6–7.7	6.7–8.7	6.3–13.8				
IgM [g/l]	$\bar{x}\pm \;\text{SD}$	0.2±0.1	0.2±0.1	0.2±0.1	0.3±0.3	0.7399	0.9759	1.0000	0.2135
	Me	0.2	0.2	0.2	0.2				
	Q_1_–Q_3_	0.2–0.2	0.2–0.3	0.2–0.3	0.2–0.4				
Large peroxidase-negative leukocytes[10^9^/l]	$\bar{x}\pm \;\text{SD}$	0.37±0.18	0.30±0.09	0.25±0.12	0.22±0.15	0.4639	0.5079	0.0684	0.3139
	Me	0.37	0.27	0.23	0.20				
	Q_1_–Q_3_	0.22–0.45	0.27–0.35	0.16–0.33	0.13–0.23				
Large peroxidase-negative leukocytes [%]	$\bar{x}\pm \;\text{SD}$	11.6±5.9	8.1±3.1	5.2±1.2	4.0±1.9	0.1930	0.0339	**0.0047**	0.0209
	Me	10.75	7.9	5.4	3.4				
	Q_1_–Q_3_	6.9–16.85	7.4–9.2	4.0–6.2	2.9–4.2				
Leukocytes [10^9^/l]	$\bar{x}\pm \;\text{SD}$	4.15±4.45	4.35±3.25	4.94±1.65	5.40±1.66	0.3622	0.4483	0.0277	0.0330
	Me	2.84	3.61	4.34	5.77				
	Q_1_–Q_3_	2.48–4.08	2.69–4.23	3.89–6.46	4.26–6.66				
Lymphocytes [10^9^/l]	$\bar{x}\pm \;\text{SD}$	0.66±0.66	0.58±0.34	1.93±1.09	1.80±1.07	0.2642	0.8010	**0.0019**	**0.0015**
	Me	0.38	0.49	1.79	1.46				
	Q_1_–Q_3_	0.30–0.68	0.45–0.59	1.15–2.33	1.08–2.07				
Lymphocytes [%]	$\bar{x}\pm \;\text{SD}$	20.5±17.0	16.0±8.9	37.0±12.3	33.6±15.5	1.0000	0.6498	**0.0047**	**0.0047**
	Me	12.2	14.9	38.2	34.1				
	Q_1_–Q_3_	9.0–36.3	9.3–21.7	27.5–43.2	25.3–43.8				
Monocytes[10^9^/l]	$\bar{x}\pm \;\text{SD}$	0.54±0.26	0.56±0.21	0.42±0.19	0.54±0.25	0.7241	0.2428	0.1159	0.3465
	Me	0.54	0.47	0.39	0.46				
	Q_1_–Q_3_	0.35–0.63	0.39–0.66	0.23–0.49	0.38–0.64				
Monocytes [%]	$\bar{x}\pm \;\text{SD}$	16.2±6.8	15.5±6.3	8.2±1.9	10.1±3.0	0.8403	0.1014	**0.0030**	0.0131
	Me	14.5	16.2	8.6	9.6				
	Q_1_–Q_3_	13.2–20.5	10.8–19.0	6.1–9.8	7.9–11.3				
NK cells [%]	$\bar{x}\pm \;\text{SD}$	17.5±15.4	32.6±23.8	12.2±5.1	17.2±10.3	0.1179	0.2703	0.1549	0.0262
	Me	14.0	27.0	9.0	13.0				
	Q_1_–Q_3_	8.2–22.0	7.1–52.5	8.6–13.2	10.2–23.5				
Neutrophils [10^9^/l]	$\bar{x}\pm \;\text{SD}$	2.54±4.24	2.95±3.13	2.08±0.68	2.67±1.31	0.1129	0.2869	0.1520	0.2489
	Me	1.40	2.38	2.03	2.06				
	Q_1_–Q_3_	0.95–2.08	1.34–2.84	1.62–2.29	1.95–3.29				
Neutrophils [%]	$\bar{x}\pm \;\text{SD}$	50.9±20.8	61.1±14.7	44.1±13.2	48.2±15.2	0.0908	0.6498	0.1005	0.0192
	Me	54.1	66.8	44.6	51.3				
	Q_1_–Q_3_	39.8–59.4	44.2–68.3	32.7–53.9	38.6–54.2				

Bold type denotes statistically significant results at 0.0125 with the Bonferroni correction;

$\bar{x}$- mean, SD -standard deviation; Me—median;Q_1_- lower quartile;Q_3_ -upper quartile; *P*1, *P*2, *P*3, *P*4—significance levels:

*P*1 -significance level in the Mann-Whitney *U* test used to compare immunological parameters in females and males with multiple myeloma on the day of the clinic discharge;

*P*2—significance level in the Mann-Whitney *U* test used to compare immunological parameters in females and males with multiple myeloma on the 29th day after discharge;

*P*3—significance level in the Wilcoxon’s signed-rank test used to compare immunological parameters in females with multiple myeloma on the day of the clinic discharge and on the 29th day after discharge;

*P*4—significance level in the Wilcoxon’s signed-rank test used to compare immunological parameters in males with multiple myeloma on the day of the clinic discharge and on the 29th day after discharge;

*—too little data to perform the analysis

No statistically significant differences were observed in the analysed immunological parameters between the studied females and males with MM on the day of the clinic discharge and on the 29th day after discharge.

Among the studied females statistically significant differences on the 29th day after the clinic discharge compared to the day of the clinic discharge were observed in 8 immunological parameters. These differences included a reduction in: the percentage of CD4(+) T lymphocytes in total lymphocyte count, from 33.5% to 10.9%, the percentage of large peroxidase-negative leukocytes in total leukocyte count, from 11.6% to 5.2%, and the percentage of monocytes in total leukocyte count, from 16.2% to 8.2%, and at the same time, an increase in the percentage of CD19(+) B lymphocytes in total lymphocyte count, from 1.2% to 10.9%, eosinophil count, from 0.03×10^9^/l to 0.26×10^9^/l, the percentage of eosinophils in total leukocyte count, from 0.9% to 5.2%, lymphocyte count, from 0.66×10^9^/l to 1.93×10^9^/l, and the percentage of lymphocytes in total leukocyte count, from 20.5% to 30.7%.

Among the studied males statistically significant differences on the 29th day after the clinic discharge compared to the day of the clinic discharge were observed in 6 immunological parameters. These differences included statistically significant increase in: the percentage of CD19(+) B lymphocytes in total lymphocyte count, from 0.6% to 11.1%, eosinophil count, from 0.01×10^9^/l to 0.24×10^9^/l, the percentage of eosinophils in total leukocyte count, from 0.1% to 4.5%, IgG concentration, from 6.8 g/l to 19.8 g/l, lymphocyte count, from 0.58×10^9^/l to 1.80×10^9^/l, and the percentage of lymphocytes in total leukocyte count, from 16.0% to 33.6%.

## Discussion

### Characteristics of the studied patients

The age of the studied patients was typical of the patients with MM in general [7] since both mean age and median age of females and males were higher than 50 years.

The studied females and males were slightly older than females and males with MM studied by Wichert et al. [8], but younger than females and males described by other authors [9–16]. Age of the patients described in case reports was highly diverse [15, 17–21].

No statistically significant differences between the studied females and males in the length of time since the diagnosis until the first transplantation and in the length of time since the diagnosis until the recent transplantation was most probably the effect of similar access to treatment and the application of similar procedures.

No information was found in the literature whether the lack of statistically significant differences between females and males in the length of time since the diagnosis until the first transplantation and the length of time since the diagnosis until the recent transplantation observed in the current study was also the case in the other populations of MM patients studied so far. Although Posch et al. [22] emphasised in the title of their article the gender-specific aspects in patients with MM, they presented a median length of time since the diagnosis until the transplantation for all the studied patients, irrespective of gender. Also Htut et al. [23], Chakraborty et al. [24], Sweiss et al. [25], Dhakal et al. [26], and Alegre et al. [27] presented the length of time since the diagnosis until the first transplantation irrespective of gender.

The median length of time since the diagnosis until the first transplantation in the studied female and male patients was high. Higher median length of time since the diagnosis until the first transplantation, 27 months, was reported only by Chakraborty et al. [24] in a subgroup of female and male patients classified as having received a delayed transplantation. In the subgroup of patients who received early transplantation [24], this length of time was 6 months. The median length of time since the diagnosis until the first transplantation was 7.5 months in Austrian patients studied by Posch et al. [22], 8 months in a group of non-African American patients and 10 months in a group of African American patients studied by Sweiss et al. [25], and 12 months in American patients studied by Dhakal et al. [26]. In Spain, the median length of time since the diagnosis until the first transplantation was 17 months in patients of both genders [27], that is the same as in the studied females, but less than in the studied males. In American patients who received second HSCT, the median length of time until the second transplantation was 7 or 8 months depending on the type of the second transplantation [23].

It is worth mentioning that more and more often the vast majority of MM patients receive the transplantation within one year. As many as 87% of patients described by Htut et al. [23], and 91% of patients aged up to 50 years and 73.3% of patients older than 70 years studied by Dhakal et al. [28] received the first transplantation within one year from the diagnosis.

The higher percentage of the studied males than females who lived more than four years after the first transplantation is in line with the results obtained by Posch et al. [22] which showed higher percentage of males, 71.8%, than females, 65.3%, who lived four years after the transplantation, however, it is noteworthy that the percentage of females was higher than in the studied females, whereas the percentage of males was lower than in the studied males. It is also interesting to note that Posch et al. [22] reported higher median progression-free survival as well as higher median overall survival both from the diagnosis until death and from the transplantation until death in males compared to females.

The Haematology and Bone Marrow Transplantation Department of the Clinical Hospital of the Poznan University of Medical Sciences is the only medical centre of this kind in the Wielkopolskie region, therefore the majority of the studied patients lived in that part of Poland.

### The immune reconstitution

In the current literature five articles were found in which the changes of immunological parameters in female and male patients with MM after autologous HSCT were analysed [8, 29–32] and only one after allogeneic transplantation [26]. The authors investigated changes in the immune system at various stages of the disease, including less parameters than in the current study, and they presented the results for the whole studied population irrespective of gender. None of the authors investigated the influence of gender on immunological parameters.

The timepoints at which immunological parameters were assessed in the current study, that is on the day of the clinic discharge and on the 29th day after discharge, were the most similar to those applied in the study by Wichert et al. [8]. The authors [8] analysed changes in leukocyte count and in the percentages of lymphocytes, monocytes and eosinophils in total leukocyte count in 6 females and 8 males after autologous HSCT on the 13th day, the 15-20th day and more than six weeks after the transplantation. From among these three terms it is reasonable to assume that the 15-20th day after the transplantation corresponds to the day of the clinic discharge in the current study, whereas more than six weeks after the transplantation corresponds to the 29th day after discharge. The patients studied by Wichert et al. [8] on the 15-20th day after the transplantation were characterised by lower leukocyte count, 3.4x10^9^/l, lower percentage of monocytes, 12.1%, and higher percentage of lymphocytes, 22.8%, than the studied females and males on the day of the clinic discharge. The percentage of eosinophils, 0.13%, was lower than in the studied females but higher than in the studied males on the day of the clinic discharge. More than six weeks after the transplantation the patients studied by Wichert et al. [8] were characterised by higher percentage of lymphocytes, 39.5%, and lower percentage of monocytes, 3.8%, and eosinophils, 4.16%, than the studied females and males on the 29th day after discharge. Leukocyte count in the patients studied by Wichert et al. [8], 5.4x10^9^/l, was the same as in the studied males but higher than in the studied females on the 29th day after discharge.

Most probably, the same modality of MM patients which is identical in both females and males [33–35], caused no statistically significant influence of gender on any of the analysed immunological parameters on the day of the clinic discharge. This might have resulted in almost the same influence on the immune reconstitution reflected in the mean values of the immunological parameters in the studied females and males. Moreover, one of the conditions on which either a female or a male patient may be discharged from the clinic is neutrophil count higher than 1.0x10^9^/l [33] or preferably in the reference range, that is between 1.8 and 7.0x10^9^/l [36]. Haemoglobin concentration should be higher than 10.0 g/dl and platelet count should be higher than 25.0x10^9^/l [33]. Both female and male patient should also tolerate food and oral medications well, and should drink 2–3 litres of fluids a day. Their body temperature should not be raised and they should not lose weight [33].

Statistically significant differences in 8 immunological parameters observed in the studied females and in 6 immunological parameters observed in the studied males on the day of the clinic discharge in comparison to the 29th day after discharge indicate similar but not the same course of the immune reconstitution in patients of both genders during the 29 days after the clinic discharge. During this period the main changes, observed both in females and males, included statistically significant increase in: eosinophil, leukocyte and lymphocyte count, the percentage of eosinophils and lymphocytes in total leukocyte count, and the percentage of CD19(+) B lymphocytes in total lymphocyte count, together with a reduction in the percentages of large peroxidase-negative leukocytes and monocytes in total leukocyte count. Additionally a reduction in the percentage of CD4(+) T lymphocytes in total lymphocyte count was found only in females, whereas increase in IgG concentration was found only in males. The observed changes may result not only from genetic and hormonal differences between females and males [37, 38], but also from the considerable differences in dietary intake of both females and males during their stay at home compared to their stay in the hospital.

## Conclusions

1. Statistically significant differences in 8 immunological parameters in the studied females and in 6 immunological parameters in the studied males on the day of the clinic discharge in comparison to the 29th day after discharge imply that gender may play a role in the immune reconstitution.
2. The current study shows the need to revise up-to-date knowledge and practice referring to the influence of gender on the immune response at various stages of MM treatment and the need to analyse the parameters in females and males separately.
3. In order to explain the observed changes in the immune system according to gender, further research should be carried out on a larger population. This would most probably make it possible to find their clinical application.

## Supporting information

S1 Data (XLSX)

## References

[1] Gil L. Infectious complication after allogeneic stem cell transplantation. Acta Haematol Pol 2010; 41(3):363–370.

[2] Gil L. Viral infections after stem cell transplantation. Acta Haematol Pol 2013; 44(3):245–250. doi: 10.1016/j.achaem.2013.07.009

[3] Karakulska-Prystupiuk E, Dwilewicz-Trojaczek J. Disorders of the immunity reconstitution after haematopoietic stem cell transplantation. Onkol Dypl 2016; 6:1–6.

[4] Piekarska A. The influence of the immune reconstitution on anti-infectious immunity in patients after allogeneic haematopoietic stem cell transplantation. PhD thesis, Akademia Medyczna, Gdańsk 2008.

[5] Pruchniewski Ł, Kwiatek M, Gil L. Infectious complications in a course of multiple myeloma. Pol Merk Lek 2018; 45(270):251–255. 30693912

[6] Piątkowska-Jakubas B, Mensah-Glanowska P, Hawrylecka D, Balana-Nowak A, Zdzilowska E, Skotnicki B. Immune reconstitution after autologous hematopoietic cell transplantation. Acta Hematol Pol 2009; 40(4):867–876.

[7] Giannopoulos K, Jamroziak K, Usnarska-Zubkiewicz L et al. Recommendations of Polish Myeloma Group concerning diagnosis and therapy of multiple myeloma and other plasmacytic dyscrasias for 2018/2019. Acta Haematol Pol 2018; 49(4):157–206.

[8] Wichert S, Pettersson A, Hellmark T, Johansson Å, Hansson M. Phagocyte function decreases after high-dose treatment with melphalan and autologous stem cell transplantation in patients with multiple myeloma. Exp Hematol 2016; 44(5):342–351. doi: 10.1016/j.exphem.2016.01.002 26774385

[9] Abruzzese MP, Bilotta MT, Fionda C, Zingoni A, Soriani A, Vulpis E et al. Inhibition of bromodomain and extraterminal (BET) proteins increases NKG2D ligand MICA expression and sensitivity to NK cell-mediated cytotoxicity in multiple myeloma cells: role of cMYC-IRF4-miR-125b interplay. J Hematol Oncol 2016; 9(134):1–19. doi: 10.1186/s13045-016-0362-2 27903272 PMC5131470

[10] Andres M, Fellerc A, Arndt V. Trends of incidence, mortality, and survival of multiple myeloma in Switzerland between 1994 and 2013. Cancer Epidemiol 2018; 53:105–110. doi: 10.1016/j.canep.2018.01.015 29414629

[11] Banach M, Jurczyszyn A, Skotnicki A. Thalidomide induced peripheral neuropathy in multiple myeloma patients. Prz Lek 2015; 72(11):629–635. 27012121

[12] Bongers ML, Hogervorst JGF, Schouten LJ, Goldbohm RA, Schouten HC, van den Brandt PA. Dietary acrylamide intake and the risk of lymphatic malignancies: The Netherlands Cohort Study on Diet and Cancer. PLoS ONE 2012; 7(6):e38016. doi: 10.1371/journal.pone.0038016 22723843 PMC3377662

[13] Dumontet C, Couray-Targe S, Teisseire M, Karlin L, Maucort-Boulch D. Real life management of patients hospitalized with multiple myeloma in France. PLoS ONE 2018; 13(5):e0196596. doi: 10.1371/journal.pone.0196596 29715281 PMC5929551

[14] Greenfield DM, Boland E, Ezaydi Y, Ross RJM, Ahmedzai SH, Snowden JA. Endocrine, metabolic, nutritional and body composition abnormalities are common in advanced intensively-treated (transplanted) multiple myeloma. Bone Marrow Transplant 2014; 49(7):907–912. doi: 10.1038/bmt.2014.63 . 24710566

[15] Skipina TM, Sane DC, Cui Ch: A plasma cell-based pericardial effusion leading to tamponade in a patient with multiple myeloma—a case report and review of the literature. Cardiovasc Pathol 2019; 40:41–46. doi: 10.1016/j.carpath.2019.02.002 . 30852296

[16] Ungerstedt JS, Watz E, Uttervall K, Johansson B-M, Wahlin BE, Näsman P et al. Autologous hematopoietic stem cell transplantation in multiple myeloma and lymphoma: an analysis of factors influencing stem cell collection and hematological recovery. Med Oncol 2012; 29(3):2191–2199. doi: 10.1007/s12032-011-0029-3 21779930

[17] Gallart T, Blade J, Martfnez-Quesada J, Sierra J, Rozman C, Vives J. Multiple myeloma with monoclonal IgG and IgD of lambda type exhibiting, under treatment, a shift from mainly IgG to mainly IgD. Immunology 1985; 55(45):45–57. 3922877 PMC1453593

[18] Greuter T, Browne M, Dommann-Scherrer C, Binder D, Renner Ch, Kapp U. IgM multiple myeloma with an extremely rare non-aggressive presentation: A case report. Oncol Lett 2016; 12(4):2801–2803. doi: 10.3892/ol.2016.5034 27698861 PMC5038373

[19] Hur M, Lee YK, Lee KM, Kim HJ, Cho HI. Pseudobasophilia as an erroneous white blood cell differential count with a discrepancy between automated cell counters: report of two cases. Clin Lab Haem 2004; 26(4):287–290. doi: 10.1111/j.1365-2257.2004.00615.x . 15279666

[20] Stuver R, Petersen A, Guerrero-Garcia TA, Matulonis U, Richardson P, Singh P. Multiple myeloma masquerading as ovarian carcinosarcoma metastases: A case report and review of the approach to multiple myeloma screening and diagnosis. Case Rep Hematol 2018: 3029650. doi: 10.1155/2018/3029650 30345126 PMC6174775

[21] Xie X, Lin Y, Cao Y, Dong W, Wu W, Zhu Y et al. Autologous stem cell transplantation in EBV-positive post-renal transplant refractory multiple myeloma: A case report and literature review. Oncol Lett 2018; 15(5):7207–7214. doi: 10.3892/ol.2018.8237 29731882 PMC5921225

[22] Posch D, Rabitsch W, Wohlfarth P, Leiner M, Porpaczy E, Drach J et al. Gender-specific aspects in patients with multiple myeloma undergoing autologous stem cell transplantation: A single-center experience. Oncology 2017; 93(5):295–301. doi: 10.1159/000478265 28803241

[23] Htut M, D’Souza A, Krishnan A, Bruno B, Zhang MJ, Fei M et al. Autologous/allogeneic hematopoietic cell transplantation versus tandem autologous transplantation for multiple myeloma: comparison of long-term postrelapse survival. Biol Blood Marrow Transplant 2018; 24(3):478–485. doi: 10.1016/j.bbmt.2017.10.024 29079457 PMC5826888

[24] Chakraborty R, Muchtar E, Kumar SK, Buadi FK, Dingli D, Dispenzieri A et al. Elevated pre-transplant C-reactive protein identifies a high-risk subgroup in multiple myeloma patients undergoing delayed autologous stem cell transplantation. Bone Marrow Transplant 2018; 53(2):155–161. doi: 10.1038/bmt.2017.228 29131152

[25] Sweiss K, Oh A, Calip GS, Rondelli D, Patel P. Superior survival in African American patients who underwent autologous stem cell transplantation for multiple myeloma. Cl Lymph Myelom Leuk 2019; 19(8):506–511. doi: 10.1016/j.clml.2019.04.019 31231013

[26] Dhakal B, Brazauskas R, Lara CA, Hari P, Pasquini M, D’Souza A. Monocyte recovery at day 100 is associated with improved survival in multiple myeloma patients who undergo allogeneic hematopoietic cell transplantation. Bone Marrow Transplant 2016; 51(2):297–299. doi: 10.1038/bmt.2015.244 26457907 PMC4956087

[27] Alegre A, Díaz-Mediavilla J, San-Miguel J, Martínez R, García Laraña J, Sureda A et al. Autologous peripheral blood stem cell transplantation for multiple myeloma: a report of 259 cases from the Spanish Registry. Bone Marrow Transplant 1998; 21(2):133–140. doi: 10.1038/sj.bmt.1701062 . 9489629

[28] Dhakal B, Nelson A, Guru Murthy GS, Fraser R, Eastwood D, Hamadani M et al. Autologous hematopoietic cell transplantation in patients with multiple myeloma: effect of age. Cl Lymph Myelom Leuk 2016; 17(3):165–72. doi: 10.1016/j.clml.2016.11.006 28159578

[29] Antlanger M, Dust T, Reiter T, Böhm A, Lamm WW, Gornicec M et al. Impact of renal impairment on outcomes after autologous stem cell transplantation in multiple myeloma: a multi-center, retrospective cohort study. BMC Cancer 2018; 18(1):1008. doi: 10.1186/s12885-018-4926-0 30342509 PMC6195957

[30] Huang TCh, Huang SY, Yao M, Lin CY, Hwang WL, Gau JP et al. Autologous stem cell transplantation in multiple myeloma: Post-transplant outcomes of Taiwan Blood and Marrow Transplantation Registry. J Formos Med Assoc 2019; 118(1 Pt 2):471–480. doi: 10.1016/j.jfma.2018.07.020 . 30119948

[31] Kopińska A, Krawczyk‐Kulis M, Dziaczkowska‐Suszek J, Bieszczad K, Jagoda K, Kyrcz-Krzemien S. The importance of the number of transplanted cells with dipeptidyl peptidase‐4 expression on the haematopoietic recovery and lymphocyte reconstitution in patients with multiple myeloma after autologous haematopoietic stem‐cell transplantation. Hematol Oncol 2017; 35(2):225–231. doi: 10.1002/hon.2267 . 28620928

[32] Medeni SS, Acar C, Olgun A, Acar A, Seyhanlı A, Taskıran E et al. Can neutrophil to lymphocyte ratio, monocyte to lymphocyte ratio, and platelet to lymphocyte ratio at day +100 be used as a prognostic marker in multiple myeloma patients with autologous transplantation? Clin Transplant 2018; 32(9):e13359. doi: 10.1111/ctr.13359 30053318

[33] Hołowiecki J. Clinical haematology. Wydawnictwo Lekarskie PZWL, Warszawa 2008.

[34] Hus I, Dmoszyńska A, Robak T. The basics of haematology. Wydawnictwo Czelej, Lublin 2019.

[35] Jamroziak K, Czyż J, Warzocha K. Plasma cell myeloma—management recommendation of the Institute of Hematology and Transfusion Medicine. Hematologia 2013; 4(4):339–357.

[36] Kopczyński Z: Reference values of blood count, biochemistry and immunochemistry. Dział Diagnostyki Laboratoryjnej Szpitala Klinicznego Przemienienia Pańskiego UM w Poznaniu, data not published, Poznań 2014.

[37] Meester I, Manilla-Muñoz E, León-Cachón RBR, Paniagua-Frausto GA, Carrión-Alvarez D, Ruiz-Rodríguez CO et al. Sexy chromosomes and the immune system: reflections after a comparative study. Biol Sex Differ 2020; 11(1):3. doi: 10.1186/s13293-019-0278-y 31937374 PMC6958619

[38] Schurz H, Salie M, Tromp G. The X chromosome and sex-specific effects in infectious disease susceptibility. Hum Genomics 2019; 13(1):2. doi: 10.1186/s40246-018-0185-z 30621780 PMC6325731

